# Easy quantitative assessment of genome editing by sequence trace decomposition

**DOI:** 10.1093/nar/gku936

**Published:** 2014-10-09

**Authors:** Eva K. Brinkman, Tao Chen, Mario Amendola, Bas van Steensel

**Affiliations:** Division of Gene Regulation, Netherlands Cancer Institute, Plesmanlaan 121, 1016 HM Amsterdam, the Netherlands

## Abstract

The efficacy and the mutation spectrum of genome editing methods can vary substantially depending on the targeted sequence. A simple, quick assay to accurately characterize and quantify the induced mutations is therefore needed. Here we present TIDE, a method for this purpose that requires only a pair of PCR reactions and two standard capillary sequencing runs. The sequence traces are then analyzed by a specially developed decomposition algorithm that identifies the major induced mutations in the projected editing site and accurately determines their frequency in a cell population. This method is cost-effective and quick, and it provides much more detailed information than current enzyme-based assays. An interactive web tool for automated decomposition of the sequence traces is available. TIDE greatly facilitates the testing and rational design of genome editing strategies.

## INTRODUCTION

Genome editing tools, such as TAL effector nucleases, zinc finger nucleases and RNA-guided endonucleases (RGENs), enable targeted mutagenesis of a selected DNA sequence in genomes of many species (1,2). In each of these methods, introduction of an endonuclease with programmable sequence specificity into a pool of cells leads to a precisely defined DNA double-strand break (DSB), which, when repaired by non-homologous end-joining, results in a mixture of unaltered and mutated DNA. The latter consists primarily of a diversity of short deletions and (more rarely) short insertions that are centered round the break site (3–5). Cells with a mutation of interest then need to be cloned in order to establish a stable mutant line.

In order to implement this approach, it is usually necessary to test the efficacy of the programmable nuclease, which can vary dramatically depending on the sequence that is targeted. For example, with RGENs one typically needs to test several single-guide RNAs (sgRNAs) that are predicted to target the endonuclease to a gene of interest (6). Commonly used assays to verify the efficacy of programmable nucleases are the enzymatic Surveyor and T7 endonuclease I cleavage assays (7,8), which detect small sequence changes. These methods are, however, semi-quantitative and suffer from high background signals when sequence polymorphisms are present. More importantly, these enzymatic assays do not provide insight into the nature and the diversity of the mutations that are introduced. This information is particularly useful if one needs to establish a clonal cell line with a specific editing outcome, such as a defined deletion size that causes a specific frame shift in an open reading frame of interest, or that generates a subtle sequence change in a regulatory element. To determine the frequency of the desired editing event in the pool of cells, one can amplify the targeted genomic region by polymerase chain reaction (PCR), clone individual DNA molecules in a bacterial vector and analyze 50–100 clones by sequencing. This approach is labor-intensive, time-consuming and relatively costly. High-throughput sequencing around the induced break site (9) is a powerful alternative, but is also expensive and usually takes several weeks in most research environments.

Here, we present a simple, rapid and cost-effective strategy that accurately quantifies the editing efficacy and simultaneously identifies the predominant types of insertions and deletions (indels) in the targeted pool of cells. The method, named TIDE (Tracking of Indels by DEcomposition), requires only two parallel PCR reactions followed by a pair of standard capillary sequencing analyses. The two resulting sequencing traces are then analyzed using specially designed software that we provide as a simple web tool and as R code (both available at http://tide.nki.nl).

## MATERIALS AND METHODS

### Cell culture and transfection

K562 cells (American Type Culture Collection) were cultured in RPMI 1640 (Gibco) supplemented with 10% fetal bovine serum (FBS, HyClone), 1% penicillin/streptomycin. A pool of K562 cells stably expressing green fluorescent protein (GFP) was generated by transduction with the lentivirus construct pCCLsin.PPT.hPGK.GFP.pre (10). Because the lentiviral construct integrates randomly, the distribution of GFP expression levels is broad. This cell pool also includes cells that that were not transduced and do not express GFP at all. For transient transfection with CRISPR vectors, 1 × 10^6^ K562 cells were resuspended in Nucleofector Solution V (Lonza) with 1 μg plasmid DNA, and electroporated in an Amaxa 2D Nucleofector using program T-016. In case of LBR editing, a clonal K562 line stably transformed with Cas9 was used.

Human retinal pigment epithelial (RPE) cells were cultured in a 1:1 mixture of Dulbecco's modified Eagle's medium (Gibco) with Nutrient F12 (Gibco) supplemented with 10% FBS (HyClone), 1% penicillin/streptomycin. CRISPR vectors were transfected with 5 μl Lipofectamine 2000 Reagent (Invitrogen) and 2.5 μg plasmid DNA in 250 μl antibiotic-free medium (Gibco).

Kc167 cells were cultured in Shields and Sang M3 Insect Medium (Sigma-Aldrich) with 0.25% Bacto Peptone (BD), 0.1% Yeast Extract (BD), 5% heat-inactivated FBS and 1% penicillin/streptomycin. Note that 1 × 10^6^ cells were electroporated with 1 μg each of Cas9 and sgRNA expression plasmid using a BioRad Gene Pulser II (450 μF, 86 V).

### Constructs

For human cells, expression vector PX330 (Addgene plasmid 42230) encoding Cas9 and chimeric guide RNA was used (11). The LBR guides were cloned into expression vector pBluescript with the sgRNA cassette of PX330 and transfected into the K562 line stably transformed with Cas9. For *Drosophila* cells, Cas9 expression vector pBS-Hsp70-Cas9 (Addgene plasmid 46294) was used in combination with pU6-BbsI-chiRNA construct (Addgene plasmid 45946) (12). The sgRNAs were designed using CRISPR design (http://crispr.mit.edu/) (13) and CHOPCHOP (https://chopchop.rc.fas.harvard.edu/) (14).

The following sgRNA sequences were used:

**Table:** 

GFP guide	5′ CATGCCGAGAGTGATCCCGG 3′
NCD1 guide	5′ GGATAGTTGCAAGTATTGTT 3′
CD59 guide	5′ CAAGGAGGGTCTGTCCTGTT 3′
LMN guide	5′ GTCTGCTCGATGACACAGCT 3′
LBR guide #1	5′ GCCGATGGTGAAGTGGTAAG 3′
LBR guide #2	5′ GAAATTTGCCGATGGTGAAG 3′

For the cloning of individual DNA fragments from the edited GFP gene, PCR products were ligated in Zero Blunt vector (Invitrogen) using standard procedures.

### PCR

Genomic DNA (∼1 × 10^6^ cells) was isolated 3 days after transfection using the ISOLATE II Genomic DNA Kit of Bioline. PCR reactions were carried out with 50 ng genomic DNA in MyTaq Red mix (Bioline) according to manufacture instructions. PCR conditions were 1 min at 95°C (1×), followed by 15 s at 95°C, 15 s at 55°C and 1 min at 72°C (25–30×). The PCR products were purified using the PCR ISOLATE II PCR and Gel Kit (Bioline).

The following primer pairs spanning the target site were used (FW, forward; RV, reverse):

**Table:** 

GFP FW	5′ GCAGAAGAACGGCATCAAGGT 3′
GFP RV	5′ AGCAGCGTATCCACATAGCG 3′
NCD1 FW	5′ CCACCACCCCTCATACAAAG 3′
NCD1 RV	5′ CTGCCCAAAGGAAAAACAAA 3′
LMN FW	5′ ACATGTCGAGCAAATCCCGA 3′
LMN RV	5′ CTCTGTCTGTTTGATGCGGC 3′
LBR FW	5′ GTAGCCTTTCTGGCCCTAAAAT 3′
LBR RV	5′ AAATGGCTGTCTTTCCCAGTAA 3′

### Sanger sequencing

Purified PCR samples (∼30 ng) were prepared for sequencing using 4 μl of BigDye terminator v3.1 (Applied Biosystems) and 5 pM primer in final volume of 20 μl. PCR program: 1 min at 96°C (1×), followed by 30 s at 96°C, 15 s at 50°C and 4 min at 60°C (30×), and finishing with 1 min incubation at 4°C (1×). Samples were analyzed by an Applied Biosystems 3730xl DNA Analyzer.

### Flow cytometry

K562-GFP cells were collected 8 days after nucleofection and directly analyzed for fluorescence using a BD FACSCalibur. Viable cells were gated on size and shape using forward and side scatter. The GFP expression was measured using a 488 nm laser for excitation.

### TIDE software

TIDE code was written in R, version 3.1.1. TIDE requires as input a control sequence data file (e.g. obtained from cells transfected without RGEN Cas9), a sample sequence data file (e.g. DNA from a pool of cell treated with RGEN Cas9) and a character string representing the sgRNA sequence (20 nt). The sequencing data files (.abif or .scf format) are imported into TIDE using the R Bioconductor (15) package *sangerseqR* (version 1.0.0). Additional parameters have default settings but can be adjusted if necessary. The web interface was constructed using the *shiny* R package, with some code adapted from the Poly Peak Parser web tool (http://spark.rstudio.com/yostlab/PolyPeakParser/). The latter is a genotyping tool that can identify heterozygous short indels in sequence traces, but it cannot resolve sequences with complex indel mixtures (16).

TIDE first aligns the sgRNA sequence to the control sequence to determine the position of the expected Cas9 break site. Next, the control sequence region upstream of the break site is aligned to the experimental sample sequence in order to determine any offset between the two sequence reads. Alignments are done using standard Smith–Waterman local alignment implemented in the *BioStrings* package in Bioconductor. From here on, the software uses the peak heights for each base, as determined by the sequence analysis software provided by the manufacturer of the capillary sequencing equipment (we used 3730 Series Data Collection Software V4 and Sequencing Analysis Software V6). TIDE uses these peak heights to determine the relative abundance of aberrant nucleotides over the length of the whole sequence trace.

Note that there is a 25% chance that an identical nucleotide will be found in the composite sequence trace when compared to the wild-type sequence at the same position, since only four different nucleotides are available. This means that the average maximum aberrant sequence signal of 75% actually represents 100% of aberrant sequence trace. The plot of this aberrant sequence signal allows the user to gauge the quality of the sequence data, verify the expected cut site and interactively select the region used for decomposition.

The decomposition is conducted on a sequence segment downstream of the break site. By default it spans from *s*+5 basepairs (bp) downstream of the break to *s*+5 bp from the end of the shortest sequence read, with *s* being the maximum indel size in bp. Sequence trace models of all possible deletions and insertions of sizes {0.*n*} (*n* is by default set to 10) are constructed from the control sample trace by shifting all peaks by the appropriate number of positions to the left or right, respectively. This is done for each of the four bases, after which the vectors of the four bases are concatenated so that the decomposition is done for all bases combined. Next, the sequence trace from the mutated DNA sample is assumed to be a linear combination of the wild-type and the modeled indel traces. This combination is then resolved by standard non-negative linear modeling, for which we used the R package *nnls*. *R*^2^ is calculated to assess the goodness of fit. The *P*-value associated with the estimated abundance of each indel is calculated by a two-tailed *t*-test of the variance–covariance matrix of the standard errors. In order to account for systematic differences between the sequence trace intensities of the control and mutated DNA, the fitting parameters are then multiplied by a constant factor such that their sum equals *R*^2^.

Finally, to model insertions, the TIDE software estimates the relative frequency with which each of the four nucleotides is introduced immediately after the break site. This is done by removal of the aggregate of estimated signals of mutants that have smaller number of insertions (including non-mutated and deletions). While this can be done for all insert sizes, TIDE currently only estimates the nucleotide composition of +1 insertions, which are the most frequently observed insertions.

## RESULTS

### The TIDE method

**Figure 1.**
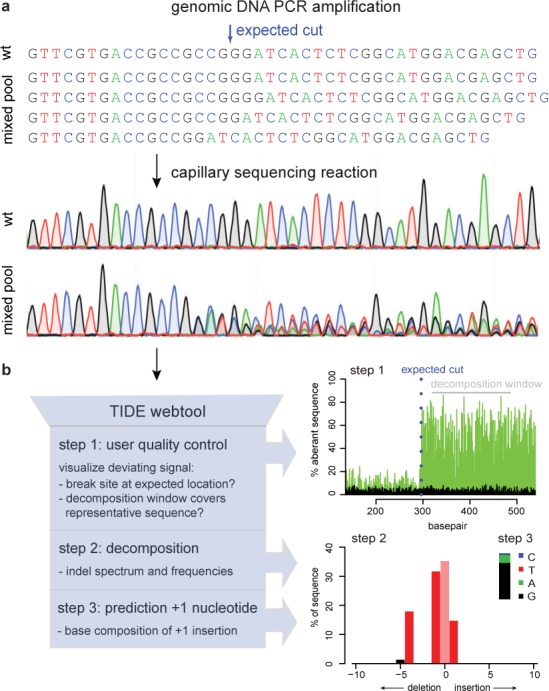
Assessment of genome editing by sequence trace decomposition. (**a**) Due to imperfect repair after cutting by a targeted nuclease, the DNA in the cell pool consists of a mixture of indels, which yields a composite sequence trace after the break site. (**b**) Overview of TIDE algorithm and output, which consists of three main steps: (1) Visualization of aberrant sequence signal in control (black) and treated sample (green), the expected break site (vertical dotted line) and the region used for decomposition (gray bar); (2) Decomposition yielding the spectrum of indels and their frequencies; (3) Inference of the base composition of +1 insertions. See main text and http://tide.nki.nl for explanation.

In the first step of TIDE, a stretch of about 500–1500 bp around the editing site is PCR amplified from genomic DNA isolated from the cell pool that was treated with the targeted nuclease. A parallel PCR amplifies the same stretch of DNA from a control cell pool lacking the nuclease or sgRNA. Both PCR products are then directly subjected to conventional capillary (‘Sanger’) sequencing, a basic technology that is available in most laboratories. In the DNA sample from the cells expressing the targeted nuclease, the sequence trace after the break site consists of a mixture of signals derived from unmodified DNA and sequences that are each shifted by a different number of nucleotides due to insertions and deletions (Figure 1a).

Based on the quantitative sequence trace data, the TIDE software first visualizes the proportion of aberrant base signals along the sequence traces in an intuitive graph (Figure 1b, step 1). This enables the user to visually inspect the sequence deviation caused by the targeted nuclease, and verify that the break site is located as expected.

Subsequently, the TIDE software decomposes the composite sequence trace into its individual components by means of multivariate non-negative linear modeling, with the control sequence trace serving as a template to model the individual indel components. This decomposition results in an estimate of the relative abundance of every possible indel within a chosen size range (Figure 1b, step 2). The software provides the *R*^2^ value as a goodness-of-fit measure, and calculates the statistical significance for each indel. In the TIDE webtool, the sequence segment used for decomposition can be interactively adjusted, which is helpful in case the sequence traces are locally of poor quality.

Finally, for insertions of a single basepair, the relative frequency of the four possible bases is deduced from the model, which is of interest if one wishes to obtain a +1 mutation of a particular sequence (Figure 1b, step 3). For longer insertions this base-calling is computationally more complicated and currently not implemented.

### *In vitro* proof of principle

**Figure 2.**
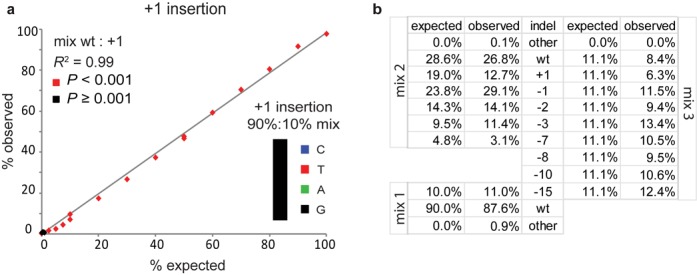
Proof-of-principle of TIDE. (**a**) A DNA fragment carrying a +1 insertion was mixed in indicated relative amounts with a corresponding wild-type DNA fragment (horizontal axis), after which the +1 insertion content was determined by TIDE (vertical axis) using the default search for indels with a size range of 0.10. Inset: relative abundance of the inserted nucleotide in a wt, +1 mix (90%:10%). See Supplementary Figure S1 for the complete decomposition results. (**b**) TIDE decomposition of various complex mixtures of wild-type DNA with DNA carrying a range of indels. See also Supplementary Figure S2a–c.

In order to test our approach, we first constructed a series of artificial samples consisting of wild-type DNA mixed with DNA carrying various indels in a broad range of relative concentrations. We then performed standard capillary sequencing and fed the resulting data into the TIDE algorithm. The constituents of the mixes could be identified and quantified with great accuracy. In a mixture of wild-type and +1 insertion DNA our algorithm was able to detect the insertion quantitatively with a sensitivity down to ∼2.5% (Figure 2a; Supplementary Figure S1), and generally predicted the correct base (Figure 2a, inset; Supplementary Figure S1). Even a −15 deletion could be reliably detected when mixed 1:10 with wild-type DNA (Figure 2b; Supplementary Figure S2a). All constituents in mixtures of wild-type DNA with either five or eight different indels were identified by TIDE (Figure 2b; Supplementary Figure S2b and c). These *in vitro* simulations show that sequence trace decomposition can accurately identify and quantify the constituent indels in a mixture.

### Application of TIDE to CRISPR/Cas9 edited DNA sequences

**Figure 3.**
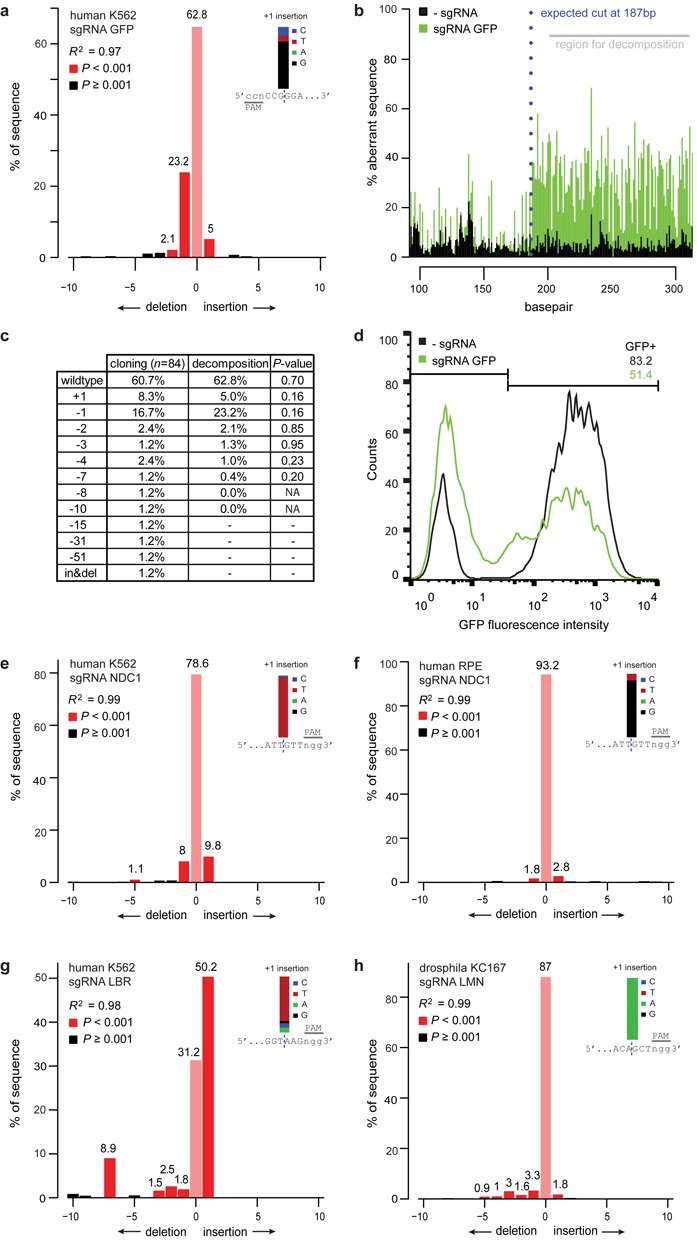
Application of TIDE to *in vivo* edited DNA sequences. (**a–d**) A pool of human K562 cells expressing GFP treated with Cas9 alone (control) and cells treated with Cas9 and a GFP targeting sgRNA (sample) were analyzed by: TIDE (a and b), sequence analysis of 84 cloned DNA fragments (c) and flow cytometry (d). (**a**) Indel spectrum determined by TIDE. Inset shows the estimated composition of the inserted base for the +1 insertion. (**b**) Aberrant nucleotide signal of the sample (green) compared to that of the control (black). Blue dotted line indicates the expected cutting site. Gray horizontal bar shows the region used for decomposition. (**c**) Comparison of indel occurrences in cloned DNA fragments (*n* = 84) to frequencies estimated by TIDE, with *P*-values according to Pearson's chi-squared test. Decomposition was limited to indels of size 0-10, hence larger indels could not be detected. (**d**) Distributions of GFP fluorescence intensities of Cas9 and Cas9+sgRNA treated cells, measured by flow cytometry. The percentage of GFP-positive cells is indicated in the top right corner within indicated histogram gate. (**e–h**) TIDE analysis of various endogenous genes (NDC1, LBR, LMN) targeted with RGENs in human cell lines (K562, RPE) and in a *Drosophila* cell line (Kc167). Insets: prediction of the inserted base for +1 insertions.

We then tested this approach on a pool of human K562 cells carrying random integrations of the GFP reporter gene. We transfected these cells with the RGEN Cas9 together with a sgRNA designed to target the GFP gene, or without the sgRNA as a control. TIDE determined that 34.2% of GFP sequences in the sgRNA-treated cell pool carried an indel, with 23.2% being a −1 deletion (Figure 3a). The composite sequence started at the expected break site (Figure 3b), confirming correct targeting by the sgRNA. The +1 insertions consisted almost exclusively of a G nucleotide on the forward strand (Figure 3a, inset), indicating that the choice of the inserted nucleotide is non-random. Sequencing of the opposite DNA strand yielded virtually identical quantitative results (Supplementary Figure S3a), indicating that the assay is highly robust.

To independently validate these results, we cloned and sequenced 84 individual DNA molecules from the same PCR product. This revealed a similar spectrum of indels, in which the frequency of each indel is generally not significantly different from the TIDE calculations (Figure 3c). However, some larger indels with frequencies below ∼2% were not significantly detected by TIDE. All +1 insertions of the individual clones consisted of a G nucleotide in the forward strand, confirming the computational inference.

All the significant mutations found by TIDE are predicted to lead to frame shifts yielding a non-functional truncated GFP protein. In agreement with this finding fluorescence-activated cell sorting analysis shows a 38.4% loss of GFP-positive cells in the pool of cells expressing the sgRNA compared to the pool lacking the sgRNA (Figure 3d). Thus, the calculation of gene editing efficiency by sequence trace decomposition is in close agreement with the observed frequency of the phenotype.

Finally, we used our approach to test different sgRNAs designed to target endogenous genes in human or *Drosophila* cell lines (Figure 3e–h). Interestingly, the results show that different sgRNAs resulted in distinct indel spectra. For example, a sgRNA targeting the *NDC1* gene produces roughly equal amounts of +1 and −1 indels, while a sgRNA targeting the *LBR* gene produces mainly +1 insertions and a few bigger deletions including a more pronounced −7 deletion. Overall, small indels (+1 and −1) appear to be the most common mutations induced by Cas9, which is in agreement with other studies (3–5). Individual indels were detected at estimated frequencies down to ∼1%. Again, the results were nearly identical when the opposite strand was sequenced (Supplementary Figure S3b and c). In addition, +1 insertions were typically dominated by one specific nucleotide, which was identical to one of the two terminal nucleotides of the break site. Which of the two neighboring nucleotides is duplicated appears to vary. For example, one sgRNA guide targeting *NDC1* resulted in different +1 insertions in K562 and RPE cells (Figure 3e and f). How the DSB repair machinery chooses the inserted bases remains to be elucidated.

## DISCUSSION

### Advantages of TIDE

Genome editing techniques like CRISPR, TALENs and ZFPs are now widely used to alter specific sequences in genomes of cultured cells. However, the efficacy and the spectrum of mutations vary greatly depending on the RGEN target site and cells used ((17,18) and this report). Hence, a fast and cost-effective approach to determine the efficacy of RGENs is essential to optimize the genome editing strategies. TIDE takes advantage of the fact that non-homologous end-joining repair of DNA DSBs leaves an indel at the break site. By decomposition of the quantitative sequence trace data, the TIDE software identifies and quantifies these indels. This allows researchers to quickly determine the efficiency of the RGENs and rationally estimate the number of cell clones that must be picked and screened in order to obtain a clonal line with a particular indel of interest.

Attractive features of TIDE are the low costs and the fact that it requires only two standard PCRs and two capillary sequencing runs. Hands-on time is therefore limited, and results can be obtained in 1 or 2 days. We found that TIDE is capable of detecting insertions and deletions with a sensitivity up to ∼1–2% across various target regions in a pool of cells. The method is highly robust, as indicated by the strong correspondence between the decomposition results from forward and reverse sequence traces. Good agreement of TIDE results with the sequence composition of a set of individually cloned DNA molecules underscores the reliability.

### Comparison to other methods

Several other methods have been used to assess genome editing efficacies. Cloning and sequencing of 50–100 individual DNA molecules provides an accurate characterization of the indel spectrum, but this is obviously more labor-intensive and 25–50 times more expensive than TIDE. Next-generation sequencing (NGS) of bulk PCR products, followed by analysis using software, such as CRISPR-GA (9), provides a highly detailed estimate of the indel spectrum, but this method is only cost-effective if a large number of samples are multiplexed; moreover, in most research institutes NGS takes several weeks.

For the creation of gene knockouts, homology-based integration of a selectable marker gene at the induced break site can facilitate the isolation of the desired clonal line (19). This approach is time-consuming and still requires moderately effective DSB induction and hence prior characterization of the efficacy of the genome editing tool is desirable.

Finally, assays that employ mismatch-detecting enzymes (7,8) require a similar amount of hands-on time as TIDE, but only estimate the overall mutation frequency and do not characterize the spectrum of indels. Moreover, polymorphisms in the vicinity of the break site are expected to cause high background signals in these enzymatic assays, unlike TIDE. Thus, TIDE offers a cost-effective and accurate alternative strategy for the rapid testing of genome editing efficacy.

### Potential limitations of TIDE

Naturally, the reliability of TIDE depends on the purity of the PCR products and the quality of the sequence reads. Decomposition results with a low *R*^2^ must be interpreted with caution. As a rule of thumb, we recommend to aim for a background signal of aberrant sequences before the break site <10% (both control and test sample), and *R*^2^ > 0.9 for the decomposition result. Sequencing of the opposite strand is recommended to confirm the results. Highly repetitive sequences around the target site may in some instances hamper the decomposition. Incorrect alignments can be detected when the quality plot shows an aberrant sequence signal that is not located at the expected break site. The decomposition window can be adjusted in order to avoid repetitive regions.

### Additional applications

While we demonstrated the utility here for Cas9-based mutagenesis, TIDE should also be applicable to other genome editing tools that are based on targeted DSB induction (1,2). Because of the quantitative nature of the results, TIDE may also be used to study mechanisms of DSB repair. For example, we observed that the spectrum of insertions and deletions varies among various target sites and cell types. It will be interesting to employ TIDE to investigate how DSB repair is affected by sequence context or local chromatin environment. Another application would be to determine differential effects of various indels at a gene of interest on cellular fitness. With TIDE, the relative abundance of indels can be followed over time in a growing population of cells treated with RGENs. An increase or decrease of wild-type sequence or particular indel over time could be an indication that the targeted sequence is lethal. In summary, TIDE will be a valuable tool for a broad diversity of research involving genome editing methods.

## SUPPLEMENTARY DATA

Supplementary Data are available at NAR Online.

SUPPLEMENTARY DATA
