# Supplementary material for: Easy quantitative assessment of genome editing by sequence trace decomposition
Source: Nucleic Acids Res. 2014 Oct 9;42(22):e168. doi: 10.1093/nar/gku936 (PMC4267669; doi:10.1093/nar/gku936)
Supplement: SUPPLEMENTARY DATA [file supp_42_22_e168__index.html]

Easy quantitative assessment of genome editing by sequence trace decomposition — Easy quantitative assessment of genome editing by sequence trace decomposition — SUPPLEMENTARY DATA 

# Easy quantitative assessment of genome editing by sequence trace decomposition

## SUPPLEMENTARY DATA

**Files in this Data Supplement:**

- SUPPLEMENTARY DATA
