# Supplementary material for: Easy quantitative assessment of genome editing by sequence trace decomposition
Source: Nucleic Acids Res. 2014 Oct 9;42(22):e168. doi: 10.1093/nar/gku936 (PMC4267669; doi:10.1093/nar/gku936)
Supplement: SUPPLEMENTARY DATA [file supp_gku936_nar-02521-met-k-2014-File005.zip › instructions.html]

### Purpose

This is a web tool to rapidly assess genome editing by CRISPR-Cas9 of a target locus determined by a guide RNA (sgRNA). Based on the quantitative sequence trace data from two standard capillary sequencing reactions the TIDE software quantifies the editing efficacy and identifies the predominant types of insertions and deletions (indels) in the DNA of a targeted cell pool. See *Brinkman et al, ##* for a detailed explanation and examples.

  

### Instructions

#### 1. Upload Data:

Enter a 20nt ('5-'3) DNA character string representing the used sgRNA guide sequence immediately upstream of the PAM sequence (PAM not included). Numbers and other invalid (non-IUPAC) DNA characters will be automatically removed. TIDE assumes that a dsDNA break is induced between nucleotides 16 and 17 in this sequence.
  
Next, upload the chromatogram sequence files of respectively the control sample (e.g. transfected without Cas9 or without the sgRNA) and the test sample (e.g. DNA of pool of cells treated with both Cas9 and the sgRNA).
  
We advise to sequence a stretch of DNA ~700bp enclosing the designed editing site. The projected break site should be located preferably ~200bp downstream from the sequencing start site. This region upstream of the break site is used to align the sequencing data of the test sample with that of the control sample.

**Currently, ABIF (.ab1) and SCF (.scf) files are supported.**  SCF is an open standard and several tools exist to convert other formats to SCF files.

#### 2. Enter Parameters for Analysis:

The following parameters have default settings but can be adjusted if necessary in the panel to the left by checking the 'advance settings' box.

#### Alignment window

These settings determine the window in which the control and test sequences are aligned to determine any offset between the two reads. There is usually no need to deviate from the default settings, except when long repetitive sequences are present.

**left boundary:**
By default this is set to 100, because base-calling at the start of a Sanger sequence read is often of poor quality.
  
**right boundary:**
This is automatically set at break site - 10bp

#### Decomposition window

These settings determine the sequence segment used for decomposition. The default setting is the largest window possible for the uploaded sequences:

**left boundary:**
max indel size + 5bp downstream of the break site.   
**right boundary:**
max indel size + 5bp before the end of the shortest sequence read.

The decomposition window can be adjusted if part of the sequence read is of low quality or contains repetitive sequences.

#### Indel size range

Set the maximum size of deletions and insertions to be modeled. The default value is 10.

#### P-value threshold

Significance cutoff. Any value between 0 and 1 is accepted. Default is p<0.001

#### 3. Results:

Once the data are uploaded and parameters are set, submit the data by clicking on the "update view" button and the plots will appear in the three tabs: "Quality", "Decomposition, "+1 insertion". If the settings are incorrect or too stringent, warnings or remarks will be dispayed in the "Quality and/or Decomposition" tab.

**Quality measures**: Results depend on the quality of the sequence reads. As a rule of thumb, we recommend to aim for an average aberrant sequence signal strength before the breaksite <10% (both control and test sample), and R2>0.9 for the decomposition result. Sequencing of the opposite strand is recommended to confirm the results.

### License, terms of use, privacy

This webtool and the associated R code are open source software under  GNU General Public License version 3. If you use this software for data analysis in a publication, please cite (Brinkman et al, ###). Your uploaded data are only used for the duration of the analysis session and are not stored or used for any other purpose.

### Code

The source code and R code of TIDE version 0.1 are available at  Bas van Steensel lab website.

### Contact

This web tool was developed by Eva Brinkman, Tao Chen and Bas van Steensel. For more information and to report bugs, please contact  Bas van Steensel

### Acknowledgements

**R**

R Core Team (2013). R: A language and environment for statistical computing. R Foundation for
Statistical Computing, Vienna, Austria.  www.R-project.org . R version 3.1.1.

**Biostrings**

H. Pages, P. Aboyoun, R. Gentleman and S. DebRoy. Biostrings: String objects representing
biological sequences, and matching algorithms. R package version 2.32.1.

**sangerseqR**

J.T. Hill and B. Demarest (2014). sangerseqR: Tools for Sanger Sequencing Data in R. R package version 1.0.0.

**nnls**

K. M. Mullen and I. H. M. van Stokkum. The Lawson-Hanson algorithm for non-negative least squares (NNLS). R package version 1.4.

**shiny**

RStudio and Inc. (2013). shiny: Web Application Framework for R. R package version 0.10.1.
 http://CRAN.R-project.org/package=shiny

**Poly Peak Parser**

Some code was adapted from
 http://spark.rstudio.com/yostlab/PolyPeakParser/

  
  
