# Supplementary figures and images for: Easy quantitative assessment of genome editing by sequence trace decomposition
Source: Nucleic Acids Res. 2014 Oct 9;42(22):e168. doi: 10.1093/nar/gku936 (PMC4267669; doi:10.1093/nar/gku936)

Supplementary Figure S1

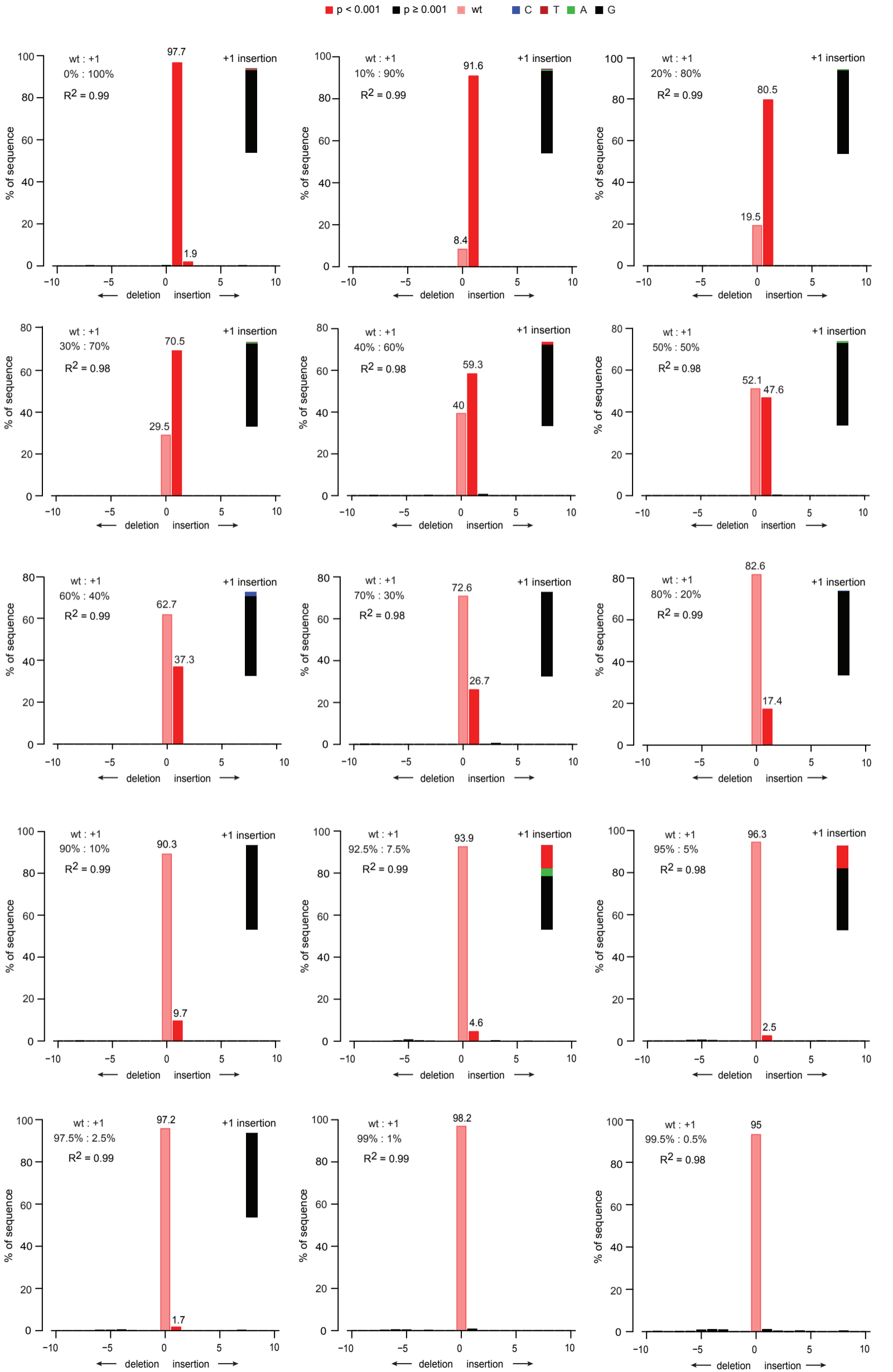

Supplement: SUPPLEMENTARY DATA [file supp_gku936_nar-02521-met-k-2014-File005.zip › fig_sup1.pdf]

Supplementary Figure S2

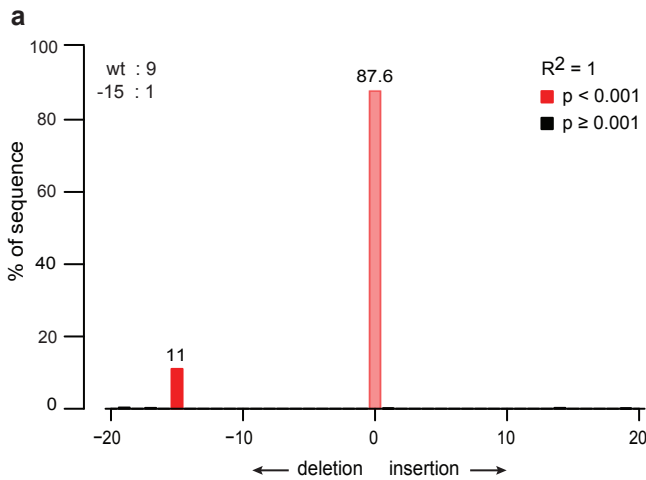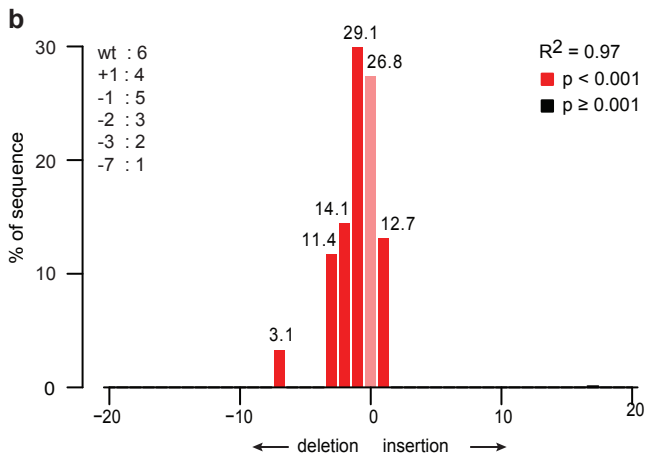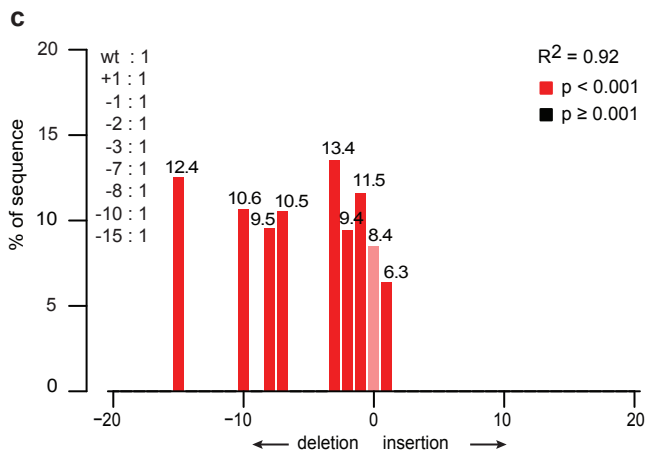

Supplement: SUPPLEMENTARY DATA [file supp_gku936_nar-02521-met-k-2014-File005.zip › fig_sup2.pdf]

# Supplementary Figure S3

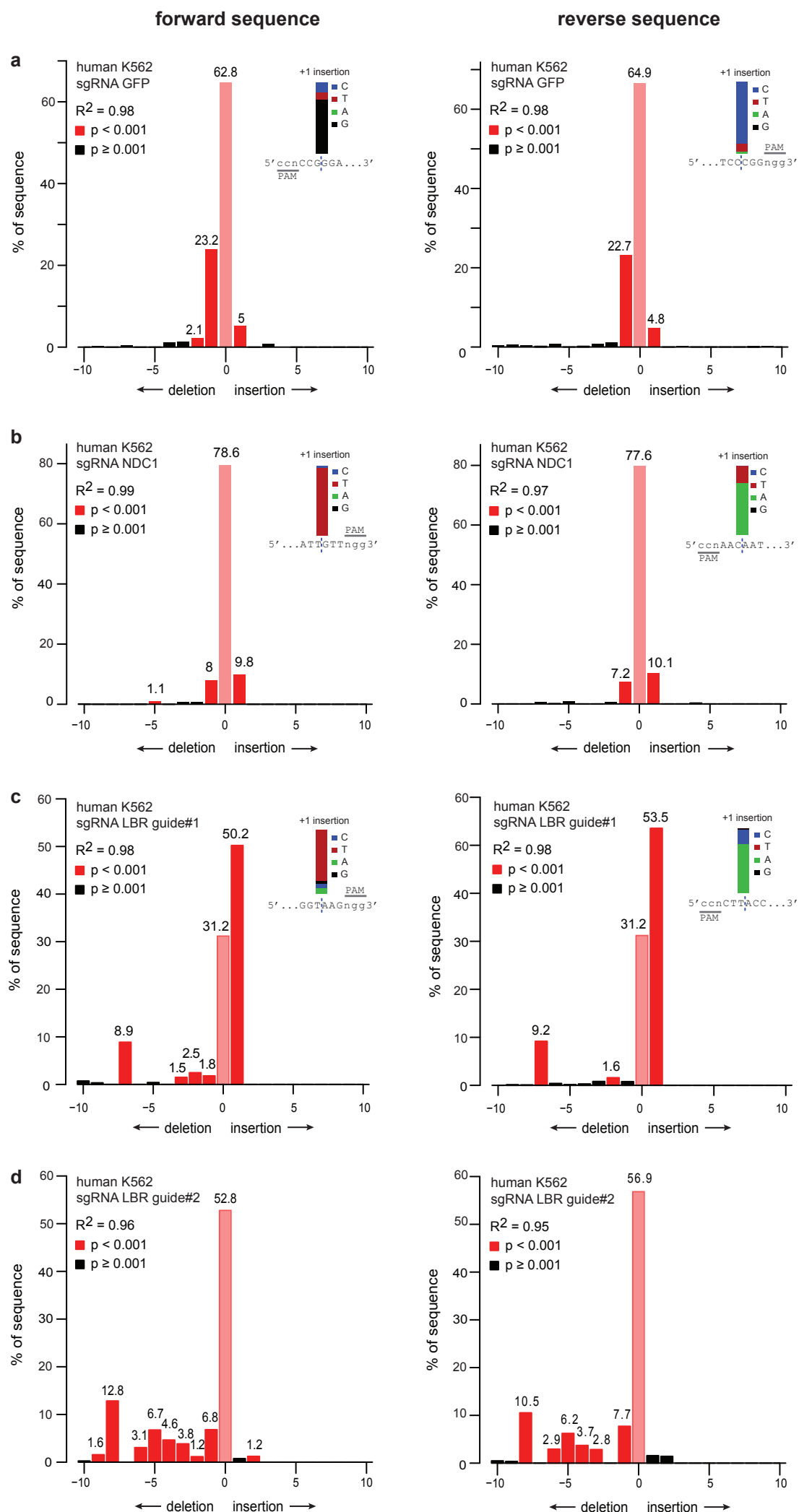

Supplement: SUPPLEMENTARY DATA [file supp_gku936_nar-02521-met-k-2014-File005.zip › fig_sup3.pdf]
